# High-throughput compound evaluation on 3D networks of neurons and glia in a microfluidic platform

**DOI:** 10.1038/srep38856

**Published:** 2016-12-09

**Authors:** Nienke R. Wevers, Remko van Vught, Karlijn J. Wilschut, Arnaud Nicolas, Chiwan Chiang, Henriette L. Lanz, Sebastiaan J. Trietsch, Jos Joore, Paul Vulto

**Affiliations:** 1 MIMETAS BV, J.H. Oortweg 19, 2333 CH, Leiden, the Netherlands; 2 Department of Molecular Cell Biology, Leiden University Medical Centre, Leiden, the Netherlands; 3 Division of Analytical Biosciences, Leiden Academic Centre for Drug Research, Leiden University, the Netherlands

## Abstract

With great advances in the field of *in vitro* brain modelling, the challenge is now to implement these technologies for development and evaluation of new drug candidates. Here we demonstrate a method for culturing three-dimensional networks of spontaneously active neurons and supporting glial cells in a microfluidic platform. The high-throughput nature of the platform in combination with its compatibility with all standard laboratory equipment allows for parallel evaluation of compound effects.

Although prevalence of central nervous system (CNS) disorders is increasing, our understanding of disease mechanisms is still very limited for most common disorders, resulting in a lack of effective treatments. For a long time, the absence of physiologically relevant *in vitro* models of the human brain was a major bottleneck in the development of new drug candidates. However, the field has immensely progressed since the availability of induced pluripotent stem cell (iPSC) techniques and accompanying differentiation protocols^1^,^2^. Current protocols account for differentiation to almost all cellular subtypes of the human brain, including a myriad of neuronal subtypes^3^,^4^,^5^,^6^,^7^, glial cells^8^,^9^,^10^ and endothelium^11^.

In parallel, the field of developmental biology has made tremendous progress in capturing the three-dimensional (3D) *in vivo* organization of stem cells, differentiated cells, and extracellular matrix (ECM) in *in vitro* grown organoids^12^,^13^,^14^. Recently, iPSC-derived neural stem cells encapsulated in ECM were shown to develop into cerebral organoids, mimicking various features of the neonatal brain, including several distinct brain regions^15^. The technique was rapidly implemented for studying brain development and the etiology of neurological disorders^16^.

With these great advances in mimicking brain physiology, the question poses itself how to implement these techniques for drug candidate and compound evaluation in a routine setting. There is a need for a system that allows assessment of efficacy and toxicity effects of libraries of compounds. Critical prerequisites of such a platform include compatibility with automated high-content imaging equipment and relatively fast readouts. Additionally, the model should utilize limited amounts of cell material per data point, while still mimicking the complexity of the human brain to the extent necessary for acquiring physiologically relevant responses.

Here we demonstrate a method for culturing 3D, ECM-embedded neuronal-glial networks in a microfluidic platform, called the OrganoPlate^®^. The OrganoPlate^®^ has a microtiter plate format comprising 96 tissue chips that can be used for 3D cell culture, co-culture, and non-invasive media exchange^17^. Human iPSC-derived neural stem cells or iPSC-derived mature neurons and astrocytes from various sources are mixed with Matrigel^®^ and seeded in these microfluidic chips. The cells form 3D networks within the chips and are characterized using a range of immunofluorescent stainings. The potential neurotoxic effects of various compounds were studied by assessing the electrophysiological activity of neurons in the network, the extent of neurite outgrowth, and the cell’s viability in response to compound treatment.

## Results

### Platform

**Figure 1:**
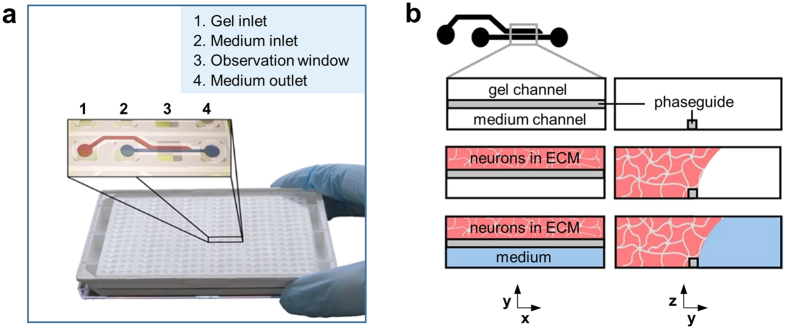
Seeding neurons and glia in the OrganoPlate^®^. **(a)** Photograph of an OrganoPlate^®^. The OrganoPlate^®^ is based on a 384-well plate and comprises a total of 96 microfluidic tissue chips. The inlay shows an artist’s impression of a single chip; each chip comprises four wells: a gel inlet (1), a medium inlet (2), an observation window (3), and a medium outlet (4) that are linked together by microfluidic channels. **(b)** Experimental outline for culturing 3D neuronal-glial networks: Cells are mixed with extracellular matrix solution (pink) and seeded in the gel channel by pipetting into the gel inlet well (1). The phaseguide is a capillary pressure barrier that restricts the gel to the gel channel. After gelation, cell culture medium (blue) is added into the medium inlet and outlet well, filling up the medium channel and enabling direct exchange of nutrients, metabolites and gasses in a membrane-free manner.

Figure 1 depicts the OrganoPlate^®^ platform. It is based on a 384-well microtiter plate format and employs coverslip-thickness glass (175 μm) for optical access. A plate comprises 96 microfluidic tissue chips, which each can be used to establish a miniaturized tissue model^17^,^18^,^19^. Each chip connects four neighboring wells: one well is used for administering the cell/ECM mixture, two wells for supplying growth medium, and a fourth well for imaging (Fig. 1a). The cell/ECM mixture is patterned in the gel channel of the tissue chip using capillary pressure barriers called phaseguides^20^. Upon gelation, the adjacent channel is filled with growth medium, enabling unimpaired exchange of nutrients, gases, and waste products (Fig. 1b). The medium inlets and outlets can be used to refresh medium and administer compounds and staining reagents to the cells.

To enable an *in vitro* model of the human brain, we optimized culture conditions for the formation of iPSC-derived 3D ECM-embedded networks of neurons and glial cells. Different types of neuronal cells, in various stages of differentiation, were used to evaluate the platform’s compatibility with neuronal culture and to explore its possible applications. Neural stem cells and early-differentiating cells were used to evaluate the option of long-term 3D neuronal differentiation, an application that is interesting for studying developmental processes, disease mechanisms, and patient specific processes. Other cells, that are already in a terminally-differentiated state upon seeding in the OrganoPlate^®^, are used to evaluate the option of short-term cultures that are convenient for applications such as compound screening. An overview of the different cell types used in this study is shown in Table 1.

### Rapid formation of three-dimensional networks

**Figure 2:**
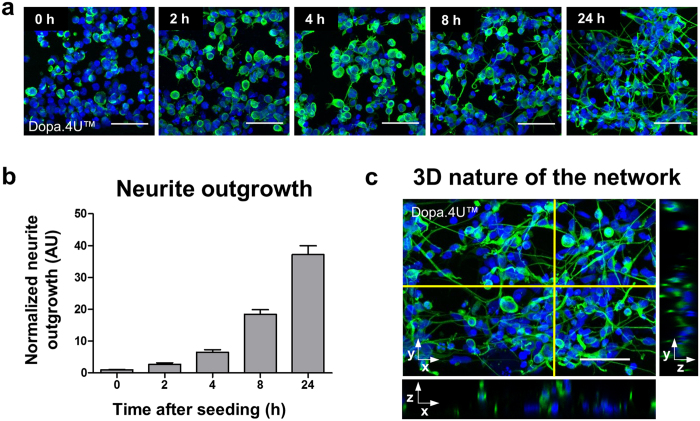
Formation of three-dimensional networks **(a)** Dopa.4U™ neurons (Axiogenesis) mixed with ECM solution were seeded in the tissue chips and allowed to form a network. Cells were fixed at different time points (0, 2, 4, 8, and 24 hours after seeding) after which the networks and nuclei were visualized by means of a β3-tubulin and DNA (DraQ5) staining. Scale bars: 50 μm. **(b)** Quantification of neurite outgrowth over time normalized to time point t = 0 h (mean ± SEM, SEM based on 200–400 cells in representative single culture chip). **(c)** Maximum projection of a Dopa.4U™ neuron culture (day 1) in the OrganoPlate^®^ stained for β3-tubulin and DraQ5. Right and bottom images show orthogonal views of the selected plain.

Neural stem cells, early differentiating cells, or mature neurons w/o astrocytes were mixed with Matrigel^®^ and seeded into the gel channel of the OrganoPlate^®^. The cells rapidly form networks within the ECM and show neuronal morphology. This is visualized in Fig. 2a, which shows network formation over time of terminally differentiated dopaminergic Dopa.4U™ neurons. The cultures were fixed at different time points after seeding and stained for neuronal marker β3-tubulin. Neurite outgrowth was quantified relative to time point zero and shows rapid network formation (Fig. 2b). The three-dimensional nature of the networks was confirmed by confocal imaging (Fig. 2c and supplementary video 1). Similar network formation was observed for co-cultures of terminally differentiated neurons and glia (iCell^®^ neurons and astrocytes, supplementary video 2).

### The OrganoPlate^®^ supports culture of various neuronal and glial subtypes

**Figure 3:**
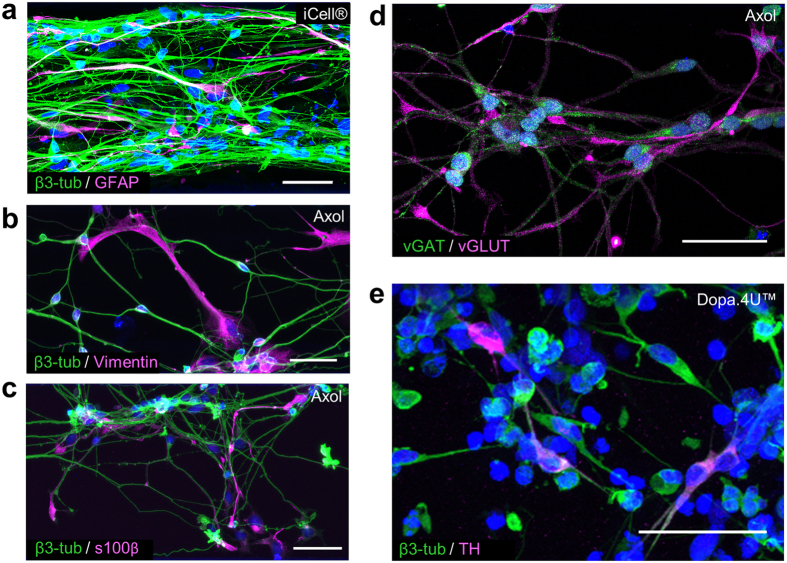
The OrganoPlate^®^ supports growth and differentiation of various cerebral cell types. **(a–c)** Maximum projections of immunofluorescent images show mixed populations of neurons and astrocytes. (**a**) A co-culture of mature iCell^®^ neurons (β3-tubulin) and astrocytes (GFAP) at day 14. (**b**,**c**) Axol Huntington neural stem cells have differentiated into neurons (β3-tubulin) and astrocytes (vimentin, s100β) after 6 weeks in the OrganoPlate^®^. Scale bars: 50 μm. **(d,e)** Maximum projections of immunofluorescent images show presence of different neuronal subtypes: (**d**) Axol neural stem cells show both glutamatergic (vGLUT) and GABAergic (vGAT) neurons after 6 weeks of differentiation, (**e**) Dopa.4U™ neurons at day 5 stained for β3-tubulin and tyrosine hydroxylase (TH). Scale bars: 50 μm.

The human brain is comprised of a myriad of cell types, including various types of neurons and glial cells. For this study, we investigated whether our 3D system supports the culture of iPSC-derived glutamatergic, GABAergic, and dopaminergic neurons. In addition, we used terminally differentiated iPSC-derived astrocytes to establish neuronal-glial co-cultures to further mimic the complexity of the human brain. Figure 3a shows a mixed population of iCell^®^ neurons and astrocytes after 2 weeks in culture. The two cell types distinctly express β3-tubulin (neurons) and glial fibrillary acidic protein (GFAP; astrocytes). Figures 3b–d show the neuronal-glial network derived from Axol iPSC-derived neural stem cells after six weeks of differentiation in the OrganoPlate^®^. A mixed population of both neurons and astrocytes was obtained as demonstrated by the expression of neuronal marker β3-tubulin and astrocyte markers vimentin and s100β (Fig. 3b,c). Moreover, the neuronal population comprises both glutamatergic (vGLUT) and GABAergic (vGAT) neurons (Fig. 3d), indicating presence of both excitatory and inhibitory cells. Figure 3e shows immunofluorescent staining of terminally differentiated Dopa.4U™ neurons (β3-tubulin) of which a subset expresses tyrosine hydroxylase (TH), the rate-limiting enzyme in dopamine synthesis.

### Spontaneous neuronal firing

**Figure 4:**
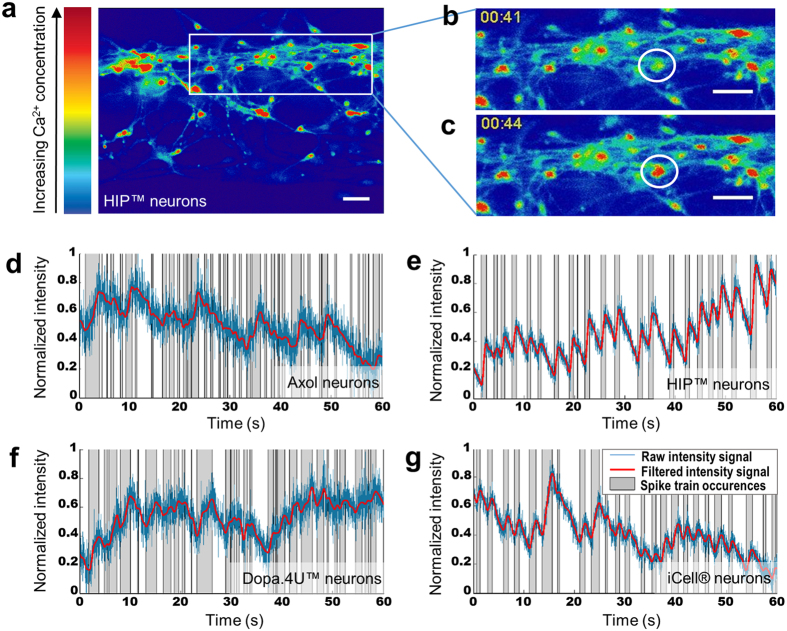
Calcium imaging shows spontaneous neuronal activity in all tested cell sources: (**a**) Example of an image acquired during calcium imaging showing HIP^TM^ neurons after 5 weeks of differentiation in the OrganoPlate^®^. The warmth of the colors corresponds to the intracellular calcium concentration. (**b**,**c**) Example of intracellular calcium fluctuations in one cell at different time points (at t = 41 s and t = 44 s). Scale bars: 50 μm. (**d**–**g**) An active cell was chosen for each of the cell sources and its intracellular fluorescence was plotted over time. The fluorescent intensity for each cell is scaled between 0 and 1, the lowest and highest fluorescent signal of that cell, respectively, and processed through a low-pass filter. Graphs show the raw intensity signal after bleach correction (blue), the filtered signal (red), and areas of activity between a local minimum and local maximum (in grey), representing different spike trains.

Spontaneous electrophysiological activity was studied in each cell source by means of calcium imaging, enabling the investigation of neuronal activity of single cells and the network as a whole. Fluo-4 AM, a calcium sensitive fluorescent dye, was loaded onto the cells and intracellular calcium fluctuations were monitored over time using high-speed imaging (Fig. 4a–c). Both the terminally differentiated neurons (Dopa.4U™ and iCell^®^) as well as the in 3D differentiated neuronal cells (Axol, HIP™) showed spontaneous electrophysiological activity. For each cell source, a representative active neuron was selected and the fluorescent signal was plotted over time (Fig. 4d–g).

### Evaluation of compound effects on 3D neuronal-glial networks

#### Electrophysiology

**Figure 5:**
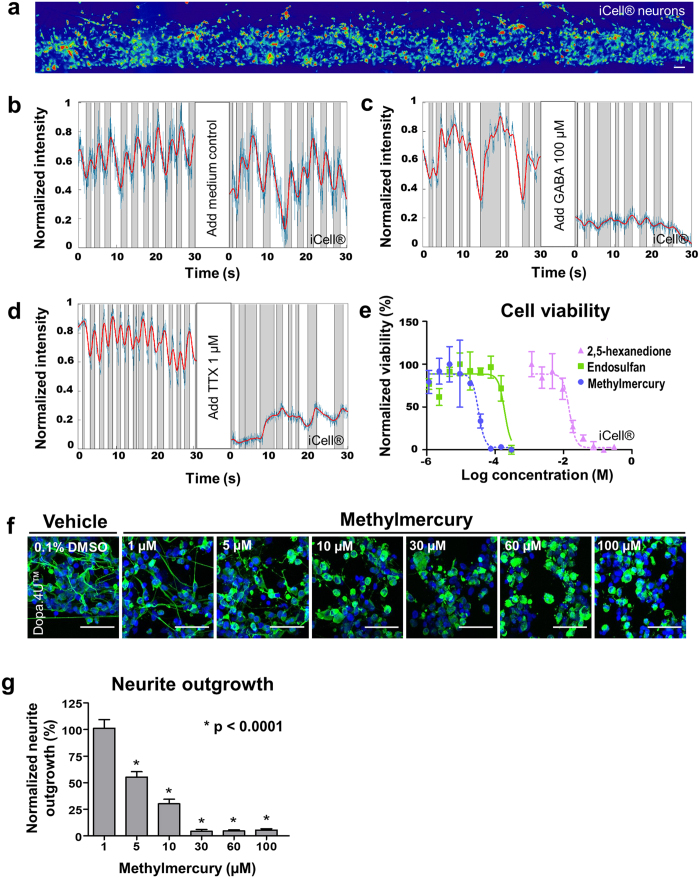
Detection of compound effects on electrophysiology, neurite outgrowth, and cell viability. (**a**) Calcium imaging recordings capture the complete network of iCell^®^ neurons (day 7) to assess electrophysiological activity before and after compound addition. The warmth of the colors corresponds to the intracellular calcium concentration. Scale bar: 50 μm. **(b–d)** Graphs show the raw intensity signal after bleach correction (blue), the filtered signal (red), and areas of activity between a local minimum and local maximum (in grey), representing different spike trains. The addition of GABA (100 μM) or TTX (1 μM) inhibits neuronal firing in iCell^®^ neurons (day 7). **(e)** Equal numbers of iCell^®^ neurons and iCell^®^ astrocytes were mixed in ECM solution and seeded in the OrganoPlate^®^. After six days in culture, the cells were exposed to various concentrations of methylmercury, endosulfan, or 2,5-hexanedione for 24 hours, after which a RealTime-Glo^TM^ assay (Promega) was used to assess cell viability. Cell viability is plotted (mean ± SD, *n* = 3) as compared to vehicle control (0.3% DMSO for methylmercury and endosulfan, medium for 2,5-hexanedione). **(f)** Dopa.4U^TM^ neurons were mixed in ECM solution and seeded in the OrganoPlate^®^ and allowed to form networks for 24 hours followed by 24-hour exposure to various concentrations of methylmercury. Cells were fixed and stained with β3-tubulin and DraQ5 to visualize neurites and nuclei, and imaged using confocal microscopy. Maximum projection images are shown. Scale bars: 50 μm. **(g)** Quantification of neurite outgrowth of neurons exposed to methylmercury shows a concentration-dependent decrease (mean ± SEM, SEM based on 200–400 cells in a representative single culture chip, Kruskal-Wallis test (non-Gaussian data set) and Dunn’s post hoc test for multiple comparison, *p < 0.0001).

To assess the applicability of our 3D cultures to high-throughput compound evaluation, we studied compound effects on electrophysiology, neurite outgrowth, and cell viability. Calcium imaging was employed to detect electrophysiological activity of neurons before and after compound addition (Fig. 5a, supplementary movies 3,supplementary movies 4,supplementary movies 5,supplementary movies 6). iCell^®^ neurons were seeded in the OrganoPlate^®^ and cultured for seven days before being exposed to GABA (100 μM) or tetrodotoxin (TTX, 1 μM). Both GABA and TTX inhibited neuronal firing of the iCell^®^ neurons, while no alteration in firing patterns was observed in the control chips (Fig. 5b–d). GABA is the main inhibitory neurotransmitter in the mature brain, but acts excitatory during neuronal development^21^,^22^. GABA’s inhibitory effect on the 3D iCell^®^ neuronal cultures in the OrganoPlate^®^ demonstrates once more that the network is comprised of a mature neuronal population. TTX is a potent neurotoxin present in puffer fish and is known to block voltage-gated sodium channels and inhibit action potential firing^23^.

#### Cell viability

Next, we assessed cell viability in a co-culture of neurons and astrocytes following exposure to various concentrations of three neurotoxic compounds. Equal numbers of iCell^®^ neurons and astrocytes were mixed with Matrigel^®^ and cultured in the OrganoPlate^®^ for six days, after which the cells were exposed to the compounds for 24 hours. A RealTime-Glo™ Cell Viability assay was employed to measure the reducing potential, and thus the viability, of the cells. All three well-established neurotoxicants tested (methylmercury^24^, endosulfan^25^, and 2,5-hexanedione^26^) reduced cell viability in a concentration-dependent manner (Fig. 5e).

#### Neurite outgrowth

Furthermore, we assessed neurite outgrowth to detect compound toxicity. Dopa.4U™ neurons were seeded in the OrganoPlate^®^ and allowed to form networks for 24 hours. The cells were subsequently exposed to a concentration range of methylmercury, a neurotoxic compound known to induce oxidative stress^24^. After 24-hour exposure, the cultures were fixed and the neuronal networks were visualized by means of a β3-tubulin staining. We observed a significant decrease in average neurite length upon increasing concentrations of methylmercury (Fig. 5f,g).

## Discussion

In this paper we introduced a method for parallel culture and testing of 3D neuronal-glial networks. The networks were grown in microfluidic channels and were comprised of iPSC-derived neurons and astrocytes from various commercial sources. The 3D nature of these cultures is an essential prerequisite for current day *in vitro* modelling, as many essential cellular processes and characteristics, including differentiation, gene expression, cell-cell interactions, and cell-matrix interactions are different in a two-dimensional planar setting as compared to *in vivo*. This makes it difficult to extrapolate results obtained from 2D cell culture to living organisms^27^,^28^,^29^,^30^. Neurons grown in 3D culture systems were observed to show longer neurite outgrowth, improved cell survival, and different patterns of differentiation as compared to 2D monolayers^31^,^32^.

Current 3D culture systems, on the other hand, have several disadvantages. The current 3D cell culture and brain organoid systems suffer from diffusional transport limitations, large variability in seeding conditions, and complex procedures for cell seeding, medium changes, staining, and imaging^30^,^33^. The platform introduced here largely solves the handling and reproducibility issues that are associated with 3D cultures. The simultaneous seeding of 96 parallel chips diminishes well-to-well variability and the platform is user-friendly, without the need of pumps. The combination of small volumes, microfluidic gel stratification, and porous extracellular matrices prevents diffusional transport limitations. Although fluid flow is not actively induced in this system, a minimum amount of flow is inherent to a multi-reservoir microfluidic channel system, providing nutrient supply and waste metabolite removal not only through diffusion, but also through interstitial flow through the extracellular matrix. Moreover, the geometrical separation between reservoirs with media and the actual culture volume in the microfluidic channel makes exchange of media and addition of compounds or staining reagents fully non-invasive, meaning that replenishment in the microfluidic space will occur through passive leveling between reservoirs, rather than through a pipetting step. In comparison to other microfluidic culture systems, the proposed platform is fully devoid of artificial membranes, enabling an unimpaired exchange of the ECM interstitial fluid and the medium channel^17^,^20^. Clearly, the platform’s high-throughput nature and compatibility with standard laboratory equipment as well as the limited amounts of cell material needed per tissue chip enables evaluation of compounds at a variety of concentrations with various duplicates. Moreover, the platform can be easily adopted in an automated setting enabling screening of larger numbers of compounds and conditions.

The 3D nature of the neuronal-glial cultures in the OrganoPlate^®^ allows cell growth and network formation in all directions and enables cell-matrix interactions, showing increased complexity as compared to traditional 2D cultures. However, the model presented in this study does not encompass all cell types present in the human brain. Additional cell types can be added to the model to increase complexity depending on the desired application. Other glial cell types, such as oligodendrocytes and microglia, can be added to study myelination and immune processes within the CNS. Moreover, iPSC-derived cells from patients of various disorders can be differentiated in the OrganoPlate^®^ to study developmental abnormalities and disease processes.

Another option is to grow a vessel of brain microvascular pericytes and endothelial cells in the medium channel adjacent to the neuronal-glial networks and mimic the blood-brain barrier (BBB)^34^. The BBB is a highly specialized barrier that ensures a homeostatic environment for the CNS. By limiting passive diffusion of hydrophilic compounds into the CNS, the BBB hampers most drugs from entering the brain. Therefore, a model consisting of astrocytes and neurons as well as a BBB will further improve drug candidate evaluation. This permits the evaluation of compound effects within the brain, as well as the compounds’ ability to enter the brain in the first place and whether they affect BBB integrity.

Finally, personalized medicine represents the future of treating CNS disorders^35^. With most disorders resulting from different causes with each patient, it is likely that tailor-made treatments are necessary to successfully target these diseases. Using iPSC technology, our method enables culture of 96 parallel neuronal-glial networks of one specific patient and allows testing of various compounds to evaluate the best treatment option, thereby increasing treatment efficacy and reducing side effects for the patient.

Taken together, we have developed a novel method enabling high-throughput evaluation of pharmaceutical compounds on 3D networks of neurons and glia in a microfluidic platform. Using various cell types, we show that the platform is compatible with long-term differentiation of neural stem cells and progenitor cells as well as short-term culture of terminally differentiated cells that can be mixed in defined ratios. Networks are formed in a robust and time-efficient manner, and assays can be performed within seven days after cell seeding. The 3D ECM-embedded networks showed compatibility with a wide range of real-time and end-point assays, including calcium imaging, neurite outgrowth, and cell viability. The user-friendliness of the platform and its compatibility with standard laboratory equipment unlocks the full potential of 3D CNS models for compound screening and evaluation purposes. Finally, using patient derived iPSCs, this method may be used for personalized medicine selection to improve treatment of common diseases of the central nervous system.

## Methods

### Materials

The following reagents were purchased from Thermo Fisher Scientific (Waltham, MA, USA): B-27^®^ supplement 50x, Fluo-4 AM cell permeant, formaldehyde 37%, GlutaMAX™ supplement, human recombinant epidermal growth factor (EGF), MEM non-essential amino acids solution 100x, N-2 supplement 100x, and phosphate buffered saline 1x (PBS). The following reagents were purchased from Sigma-Aldrich (St. Louis, MO, USA): 2,5-hexanedione, γ-Aminobutyric acid (GABA), bovine serum albumin (BSA), dimethyl sulfoxide (DMSO), Dulbecco’s Modified Eagle Medium (DMEM), endosulfan, methylmercury, penicillin-streptomycin solution 100x, Triton™ X-100, and TWEEN^®^ 20. Matrigel^®^ was purchased from Corning (Corning, NY, USA). Fetal calf serum (FCS) was purchased from ATCC (Manassas, VA, USA). Human recombinant fibroblast growth factor basic (bFGF) was purchased from Peprotech (Rocky Hill, NJ, USA). Tetrodotoxin (TTX) was purchased from Tocris Bioscience (Bristol, UK).

### Cell culture

Four commercially available sources of neuronal and glial cell types were used for this study. Neural stem cells (Huntington’s disease patient derived) were kindly provided by Axol Bioscience (Cambridge, UK), HIP™ neurons were purchased from AMSBIO (Abingdon, UK), Dopa.4U™ neurons from Axiogenesis (Köln, Germany), and iCell^®^ neurons and astrocytes from Cellular Dynamics International (Madison, WI, USA). All neuronal cell types were cultured in media provided by the supplier with the addition of 100 U/mL penicillin and 100 μg/mL streptomicin. iCell^®^ astrocytes were cultured on Matrigel^®^ coated flasks (80 μg/mL Matrigel^®^ in DMEM, incubated for 15 minutes at 37 °C, 5% CO_2_) in DMEM supplemented with 15% FCS, 2 mM GlutaMAX™, 1% non-essential amino acid solution, 100 U/mL penicillin, 100 μg/mL streptomicin, 10 μg/mL bFGF and 10 μg/mL EGF for 4 passages before seeding in the OrganoPlate^®^. All neural cells were seeded into the OrganoPlate^®^ immediately after thawing and centrifugation.

### Seeding and cultivation of neuronal and neuronal-glial cultures in the OrganoPlate^®^

Cells were thawed and spun down according to manufacturer’s instructions. After centrifugation, the supernatant was aspirated and the cells were resuspended in 7 mg/mL Matrigel^®^ (diluted from 9.3 mg/mL Matrigel^®^ stock using cold DMEM) to reach a concentration of 25,000 cells/μL. Using a repeating pipette (Sartorius eLINE electronic pipette), 0.5–1 μL of the cell suspension was seeded in the gel channel of each tissue chip of the OrganoPlate^®^ (MIMETAS, the Netherlands). Subsequently, the OrganoPlate^®^ was placed in the incubator (37 °C, 5% CO_2_) for 20 minutes to allow gelation of Matrigel^®^. Next, 50 μL of medium (provided by the supplier with the addition of 100 U/mL penicillin and 100 μg/mL streptomycin) was added to the medium inlets, followed by adding 50 μL medium to the medium outlets and placing the OrganoPlate^®^ back into the incubator (see Fig. 1b). Half medium changes were performed twice a week by aspirating half the medium from the medium inlet and outlet wells, and adding 25 μL of fresh medium. Phase-contrast images were taken from all 96 tissue chips of the OrganoPlate^®^ 2 to 3 times per week using an ImageXpress Micro XLS microscope (Molecular Devices, Sunnyvale, CA, USA) to assess progress and consistency of the cultures.

### Immunocytochemistry

*Each step describes the procedure and volumes used for one chip. For each step of the protocol, 50* *μL of solution is added to both the medium inlet and medium outlet of a chip unless specified otherwise. The gel inlets are left empty during all procedures. All steps are performed at room temperature (RT) unless specified otherwise. Each wash step lasts 5* *minutes.* Medium was aspirated from the medium inlets and outlets of the chips and replaced with PBS to wash the cells for five minutes. PBS was aspirated and cells were fixed using 3.7% (v/v) formaldehyde in PBS for 15 minutes. The fixative was aspirated and wells were washed three times with PBS and once with 4% FCS (v/v) in PBS. Next, cells were permeabilized for 10 minutes in 0.3% (v/v) Triton X-100 in PBS and washed once with 4% FCS in PBS. Cells were blocked in 2% (v/v) FCS, 2% BSA (w/v), 0.1% (v/v) TWEEN^®^ 20 in PBS for 45 minutes and incubated with primary antibody solution (40 μL per chip; 10 μL on medium inlet, 30 μL on medium outlet) for 1 hour at RT or overnight at 4 °C. The following primary antibodies were diluted in blocking solution and used for immunocytochemistry: β3-tubulin (1:200, Abcam, AB78078), β3-tubulin (1:200, Abcam, AB18207), GFAP (1:200, Millipore, AB5804), Vimentin (1:20, Abcam, AB8978), S100β (1:100, Abcam, AB52642), vGLUT (1:1000, Abcam, AB72310), vGAT (1:750, Synaptic systems, 131011), and tyrosine hydroxylase (1:2000, Santa Cruz, H-196). Wells were washed three times with 4% FCS in PBS, followed by the addition of 40 μL secondary antibody solution (10 μL on medium inlet, 30 μL on medium outlet). The following secondary antibodies were used: Alexa Fluor^®^ goat-anti-mouse 488, Alexa Fluor^®^ goat-anti-rabbit 488, Alexa Fluor^®^ goat-anti-mouse 555, and Alexa Fluor^®^ goat-anti-rabbit 555 (Thermo Fisher, diluted 1:250 in blocking solution). After 30 minutes of incubation in the dark, wells were washed three times with 4% FCS in PBS and once with PBS. DraQ5 (Abcam, 1:1000 diluted in PBS, 10 μL on medium inlet, 30 μL on medium outlet) was added to stain the nuclei and incubated for 20 minutes. The DraQ5 solution was aspirated and replaced with PBS. High quality Z-stack images of the stained cells were obtained using a Leica TCS SP5 confocal microscope (Leica, Wetzlar, Germany).

### Calcium imaging

*Each step describes the procedure and volumes used for one chip. For each step of the protocol, 50 μL of solution is added to both the medium inlet and medium outlet of a chip unless specified otherwise. The gel inlets are left empty during all procedures.* Spontaneous neuronal activity was recorded by calcium imaging using the Fluo-4 AM dye. The medium of the selected wells was replaced with 5 μM Fluo-4 AM in medium (final concentration DMSO equalled 0.1%) and incubated for 30 minutes at RT. Next, the Fluo-4 solution was replaced with conditioned dye-free medium and incubated for 15 minutes at RT. High-speed imaging was performed to detect changes in fluorescent signal using the Leica AF6000 microscope (20x magnification, 50 Hz, binning 2 × 2). For each cell source, several active cells were analyzed and a representative recording was selected to plot the fluorescent signal over time. Calcium recordings were corrected for photo-bleaching using a bleach correction plugin in Fiji^36^. The signal of each selected cell was scaled between 0 and 1, and processed through a low-pass filter (Butterworth at 0.5 Hz, 3^rd^ order) in MATLAB^®^ (2013b and Signal Processing Toolbox, The MathWorks Inc, Natick, MA, USA). Results show the raw intensity signal after bleach correction (blue), the filtered signal (red), and areas of activity between a local minimum and local maximum (in grey), representing different spike trains (Fig. 4d–g).

### Calcium imaging compound assay

iCell^®^ neurons were loaded with Fluo-4 AM as described above. Immediately before imaging, medium was aspirated from the medium inlet and outlet wells to ensure that compounds were not significantly diluted upon addition to the microfluidic chip. The submicroliter volume of medium left inside the microfluidics itself was not aspirated to avoid affecting the cells. Spontaneous activity was recorded using the ImageXpress Micro XLS-C Confocal High-Content Imaging System (Molecular Devices, wide field mode, 20x magnification, 20 Hz) for 30 seconds. Image acquisition was halted and 50 μL of medium containing 100 μM GABA or 1 μM TTX was carefully pipetted into the medium inlet and outlet wells. Image acquisition was resumed after 30 seconds to record compound effects. The recordings made before and after compound addition were individually corrected using a bleach correction plugin in Fiji^36^. This bleach correction plugin corrects the mean intensity of all frames to the first frame of each recording. To correct for the advanced bleaching at the start of the second recording (30 seconds after compound condition), the mean intensity of all frames of the second recording was multiplied by the ratio of the mean intensity of both recordings. The bleach corrected recordings were concatenated and regions of interest were selected manually to plot the fluorescence signal of several cells over time. The signal of a representative cell was processed and plotted as described above (Fig. 5b–d).

### Neurite outgrowth

Dopa.4U™ neurons were seeded in the OrganoPlate^®^ as described above and fixed at different time points after seeding (0, 2, 4, 8, and 24 hours) according to the procedure described in the immunocytochemistry section. Neurons were stained with β3-tubulin and DraQ5 to visualize neurites and nuclei and imaged on the Leica TCS SP5 confocal microscope (Fig. 2a). Maximum projection images of nuclei only and nuclei plus β3-tubulin staining were analyzed using the neurite outgrowth application of the Molecular Devices MetaXpress software (MetaXpress 6.1). Errors in cell or neurite detection were manually corrected and values of false positives were replaced by pixel length 0. Graphs were plotted using GraphPad Prism 6 (GraphPad Software, San Diego, CA, USA) (Fig. 2b).

### Compound effects on neurite outgrowth

Dopa.4U™ neurons were seeded in the OrganoPlate^®^ as described above and allowed to form networks for 24 hours. Next, the cells were exposed to methylmercury or vehicle control (0.3% DMSO, v/v) in complete Dopa.4U™ medium for 24 hours. Cells were subsequently stained and imaged and neurite outgrowth was analyzed as described above (Fig. 5f). Neurite outgrowth in each condition was normalized to vehicle control (0.3% DMSO). Graphs were plotted and statistical analysis was performed using GraphPad Prism 6. The neurite outgrowth data set (non-Gaussian) was analyzed using a Kruskal-Wallis test and a Dunn’s post-hoc test for multiple comparisons (Fig. 5g).

### Compound effects on cell viability

iCell^®^ neurons and astrocytes were seeded in a 1:1 ratio in the OrganoPlate^®^ and cultured for 6 days after which the cells were exposed to a concentration range of neurotoxic compounds for 24 hours. For methylmercury and endosulfan the vehicle control was 0.3% v/v DMSO and for 2,5-hexanedione the vehicle control was medium. After 24 hours, the cell viability was assessed using the bio-luminescent RealTime-Glo^TM^ MT assay (Promega, Madison, WI, USA) according to manufacturer’s instructions. The luminescent signal was measured every 5 minutes using the Fluoroskan Ascent^TM^ Microplate Fluorometer (Thermo Scientific). For each chip, the medium inlet, the observation window, and the medium outlet were measured and the average value was determined. Three chips were used per condition and the normalized average and standard deviation of each condition were plotted using GraphPad Prism 6 (Fig. 5e).

## Additional Information

**How to cite this article**: Wevers, N. R. *et al*. High-throughput compound evaluation on 3D networks of neurons and glia in a microfluidic platform. *Sci. Rep.* **6**, 38856; doi: 10.1038/srep38856 (2016).

**Publisher's note:** Springer Nature remains neutral with regard to jurisdictional claims in published maps and institutional affiliations.

## Supplementary Material

Supplementary Information

Supplementary Video 1

Supplementary Video 2

Supplementary Video 3

Supplementary Video 4

Supplementary Video 5

Supplementary Video 6

## Figures and Tables

**Table 1:** Overview and characteristics of iPSC-derived cells used in this study.

Name	Supplier	Characteristics
Axol neural stem cells	Axol Bioscience	Human iPSC-derived neural stem cells derived from a Hunginton’s disease patient. Require approximately six weeks of differentiation.
HIP™ neurons	AMSBIO	Human iPSC-derived early differentiating neurons and astrocytes derived from a healthy individual. Require approximately five weeks of differentiation.
Dopa.4U™ neurons	Axiogenesis	Human iPSC-derived terminally differentiated neurons derived from a healthy individual. A subpopulation of these neurons is dopaminergic.
iCell^®^ neurons	Cellular Dynamics International	Human iPSC-derived terminally differentiated neurons derived from a healthy individual. The neurons are predominantly GABAergic and glutamatergic.
iCell^®^ astrocytes	Cellular Dynamics International	Human iPSC-derived astrocytes derived from a healthy individual.
