# Supplementary material for: High-throughput compound evaluation on 3D networks of neurons and glia in a microfluidic platform
Source: Sci Rep. 2016 Dec 9;6:38856. doi: 10.1038/srep38856 (PMC5146966; doi:10.1038/srep38856)
Supplement: Supplementary Information [file srep38856-s1.pdf]

High-throughput compound evaluation on 3D networks of neurons and glia in a microfluidic platform

Nienke R. Wevers, Remko van Vught, Karlijn J. Wilschut, Arnaud Nicolas, Chiwan Chiang, Henriette L. Lanz, Sebastiaan J. Trietsch, Jos Joore, Paul Vulto

SUPPLEMENTARY INFORMATION

Supplementary video 1 | Dopa.4U™ neurons (Axiogenesis) form 3D networks within 24 hours after seeding in the OrganoPlate®. The first frame shows a maximum projection image of Dopa.4U™ neurons (24 hours after seeding) stained with β3-tubulin (green) and DraQ5 (blue) to visualize neurites and nuclei. Subsequent frames show images taken at different depths within the gel channel of the microfluidic chip to illustrate the three-dimensional nature of the network. Images were taken using a Leica TCS SP5 confocal microscope (Leica).

Supplementary video 2 | Network formation in a co-culture of iCell® neurons and astrocytes (Cellular Dynamics International). Equal numbers of iCell® neurons and astrocytes were mixed with ECM solution and seeded in the gel channel of the OrganoPlate®. Phase contrast images were taken every 15 minutes using an ImageXpress Micro XLS microscope (Molecular Devices).

Supplementary video 3 | Calcium imaging recording of one microfluidic chip in the OrganoPlate®. iCell® neurons were seeded in ECM solution in the gel channel of the OrganoPlate® and calcium imaging was performed at day 6 to record spontaneous neuronal activity using the ImageXpress Micro XLS-C Confocal High-Content Imaging System (Molecular Devices, wide field mode). Images were corrected for photo-bleaching using a bleach correction plugin in Fiji. A minimum projection of the time lapse was made and subtracted from each frame of the recording. A grouped Z-projection was applied (group size is 10 frames) and the recording was given a time stamp in Fiji.

Supplementary video 4 | Assessment of electrophysiological activity of iCell® neurons in response to medium addition. iCell® neurons (day 7) were imaged for 30 seconds to record baseline neuronal activity. Recording was halted and medium was added to medium inlet and outlet wells of the microfluidic chip. Recording was resumed 30 seconds after medium addition.

Supplementary video 5 | Assessment of electrophysiological activity of iCell® neurons in response to GABA exposure. iCell® neurons (day 7) were imaged for 30 seconds to record baseline neuronal activity. Recording was halted and GABA (100 µM) was added to medium inlet and outlet wells of the microfluidic chip. Recording was resumed 30 seconds after compound addition.

Supplementary video 6 | Assessment of electrophysiological activity of iCell® neurons in response to TTX exposure. iCell® neurons (day 7) were imaged for 30 seconds to record baseline neuronal activity. Recording was halted and TTX (1 µM) was added to medium inlet and outlet wells of the microfluidic chip. Recording was resumed 30 seconds after compound addition.
